# Mathematical modeling in the health risk assessment of air pollution-related disease burden in China: A review

**DOI:** 10.3389/fpubh.2022.1060153

**Published:** 2022-11-23

**Authors:** Chee Yap Chung, Jie Yang, Xiaogang Yang, Jun He

**Affiliations:** ^1^Department of Chemical and Environmental Engineering, University of Nottingham Ningbo China, Ningbo, Zhejiang Province, China; ^2^Department of Mathematics, University of Hull, Hull, United Kingdom; ^3^Department of Mechanical, Materials and Manufacturing Engineering, University of Nottingham Ningbo China, Ningbo, Zhejiang Province, China

**Keywords:** air pollution, COPD, lung cancer, relative risk, temperature, health risk assessment, mathematical model

## Abstract

This review paper covers an overview of air pollution-related disease burden in China and a literature review on the previous studies which have recently adopted a mathematical modeling approach to demonstrate the relative risk (RR) of air pollution-related disease burden. The associations between air pollution and disease burden have been explored in the previous studies. Therefore, it is necessary to quantify the impact of long-term exposure to ambient air pollution by using a suitable mathematical model. The most common way of estimating the health risk attributable to air pollution exposure in a population is by employing a concentration-response function, which is often based on the estimation of a RR model. As most of the regions in China are experiencing rapid urbanization and industrialization, the resulting high ambient air pollution is influencing more residents, which also increases the disease burden in the population. The existing RR models, including the integrated exposure-response (IER) model and the global exposure mortality model (GEMM), are critically reviewed to provide an understanding of the current status of mathematical modeling in the air pollution-related health risk assessment. The performances of different RR models in the mortality estimation of disease are also studied and compared in this paper. Furthermore, the limitations of the existing RR models are pointed out and discussed. Consequently, there is a need to develop a more suitable RR model to accurately estimate the disease burden attributable to air pollution in China, which contributes to one of the key steps in the health risk assessment. By using an updated RR model in the health risk assessment, the estimated mortality risk due to the impacts of environment such as air pollution and seasonal temperature variation could provide a more realistic and reliable information regarding the mortality data of the region, which would help the regional and national policymakers for intensifying their efforts on the improvement of air quality and the management of air pollution-related disease burden.

## Air pollution-related disease burden in China

### Air pollution

Air pollution is a global health hazard, which is recognized as a major global concern. Exposure to ambient air pollution has been linked with an increased risk of morbidity and mortality, as well as is a predominant factor toward global disease burden (1). According to the Global Burden of Disease (GBD) study 2015, China was ranked among the world's 10 most populous countries for its mortality rate due to ambient air pollution (2). The GBD study 2015 also estimated that 1.1 million people who die prematurely in China and 21.8 million disability-adjusted life years (DALYs) attributable to ambient air pollution (2). As China has experienced rapid economic growth over the past few decades, the levels of energy consumption, industrial waste, motor vehicle usage, and urban development have all increased, which contribute toward a dramatically increase in ambient air pollution levels. The increase of population in the country also requires a higher level of energy consumption where coal is the primary energy utilized, thus exacerbating local ambient air quality (3).

The impact of air pollution on the disease burden in China due to industrialization, urbanization, and rapid economic growth in the country have been mentioned and studied over the past two decades. Furthermore, it is necessary to mention the economic burden that is linked to the disease burden caused by air pollution in China. In 2016, the economic loss caused by air pollution-related disease burden was estimated to be 101.39 billion US dollar, contributing to 0.91% of the total GDP in China (4).

The six major ambient air pollutants identified by the China National Ambient Air Quality Standards (GB3095-2012) and most of other countries in the world, which include particulate matter with aerodynamic diameter smaller than 2.5 μm (PM_2.5_), particulate matter with aerodynamic diameter smaller than 10 μm (PM_10_), carbon monoxide (CO), nitrogen dioxide (NO_2_), sulfur dioxide (SO_2_), and ground-level ozone (O_3_), can a potential hazard to human health. In China, PM_2.5_ is commonly considered as the main contributor to the local Air Quality Index value (5) and the major pollutant in most regions (6). However, other ambient air pollutants could potentially affect the human health when a higher exposure level is reached.

#### Particulate matter (PM_2.5_ and PM_10_)

Particulate matter (PM) is a complex heterogeneous mixture of solid and liquid particles suspended in the air, varying from a few nm to tens of μm in sizes (7). The components in ambient PM can be vary across different regions or emission sources, but most of them may include carbon, sulfates, nitrates, trace elements, and mist. The source of PM mixtures can be identified as primary emission or secondary formation processes, for example, power plant, oil refineries, residential fuel combustion and construction (7). Airborne PM has received a lot of attention in the last few decades as they can pose the greatest risk to health. They are commonly divided into PM_2.5_ (fine particle that can penetrate gas-exchange area of the lung) and PM_10_ (coarse particle that can penetrate the lower respiratory system) according to their particle size for the research analysis and their independent effects on health outcomes (8).

According to the GBD study 2010, it was reported that exposure to ambient PM_2.5_ could increase the risk of disease outcomes such as lower respiratory infections (LRI), trachea, bronchus, and lung (TBL) cancers, cerebrovascular disease, and COPD (9). The exposure to ambient PM_2.5_ in different regions was measured and collected using different methods, including surface monitor measurements, aerosol optical depth from satellites, and TM5 global atmospheric chemistry transport model (9). Furthermore, the GBD study 2010 has reported that ambient PM pollution contributed to 46,732 thousand and 29,431 thousand global DALYs in male and female populations, respectively (9), whereas it contributed to 16,068 thousand and 9,160 thousand DALYs in male and female populations, respectively, in China (10). The GBD study 2015 has showed that all mortalities related to global ambient PM pollution have increased from 3,934 thousand cases in 2005 to 4,241 thousand cases in 2015, with a total of 7.8% increase over the past 10 years (11). The GBD study 2019 revealed that one of the largest increases in risk exposure from 2010 to 2019 was ambient PM pollution (12).

#### Gaseous pollutants (CO, NO_2_, SO_2_, and O_3_)

The most common gaseous pollutants in ambient air are carbon monoxide (CO), nitrogen dioxide (NO_2_), sulfur dioxide (SO_2_), and ozone (O_3_). Although NO_2_ has a reddish-brown color, most of the gaseous pollutants are colorless and invisible, especially when they appear in ambient air. The anthropogenic emission of the gaseous pollutants is often linked to the human activities involved in industrialization and urbanization. Each of the gaseous pollutants has its unique chemical characteristics, as well as different harmful impacts on human health.

Carbon monoxide (CO), which is formed during the incomplete combustion of carbonaceous matter, has been recognized as a chemical by-product of industrialization, for example automobile exhaust and industrial processes that burn fossil fuels (13). CO is a colorless, odorless, and tasteless gas that can be harmful to human health at high concentration levels in ambient air. When a person inhales CO, it can easily bind with hemoglobin to form carboxyhemoglobin, thus resulting in a reduction of oxygen carrying capacity in the bloodstream (14). Eventually, the person could experience neurological symptoms of CO poisoning due to the high level of carboxyhemoglobin such as headache, dizziness, nausea, and confusion (14). In addition, the new studies also revealed that exposure to ambient CO is associated with increased risks of adverse health outcomes such as cardiovascular and respiratory disease mortality, neurocognitive impairment, and behavioral disorders in children (15–18).

Nitrogen dioxide (NO_2_) is commonly emitted from the anthropogenic combustion processes such as power plants and traffic sources in urban area (19). NO_2_ emissions may interfere with the formation of other air pollutants such as particulate matter and ozone (20). Previous study also showed that a short-term exposure to NO_2_ is linked to the increased risk of respiratory mortality or morbidity including respiratory inflammation and lung function impairment (21). Furthermore, it also suggested that NO_2_ exposure might be associated with total mortality and cause-specific mortality due to cardiorespiratory diseases (22).

Sulfur dioxide (SO_2_) is an irritant gas pollutant and bronchoconstrictor, which can adversely affect the respiratory system and lead to an increased risk of mortality (23). With the characteristics of acidifying effect and high reactivity of SO_2_, it can easily react with atmospheric rain to generate weak sulfuric acid, which is a key component of acid rain, thus aggravating the effect of the pollution. In addition, SO_2_ can accumulate in the atmosphere for longer period and can be spread to different locations (24). The largest contribution of SO_2_ emission is mainly due to human activity, especially from energetic sector such as power stations and combustion plants.

Ground-level ozone (O_3_), also known as tropospheric ozone, is one of the six criteria air pollutants prescribed by China National Ambient Air Quality Standards. O_3_ is a secondary pollutant which is generated from a series of complex reactions between carbon monoxide (CO), methane (CH_4_), nitrogen oxides (NO_X_) or other volatile organic compounds (VOCs) in the atmosphere in the presence of ultraviolent radiation (8, 25). Most of the CH_4_, NO_X_, and VOCs pollutants are emitted from the industrial activities and urbanization process, especially in the densely populated cities in China due to the high energy consumption required (25). The emission of O_3_ pollutant is considered as photochemical process, thus typically occurring during the warm seasons and peaks in the daytime (26). O_3_ pollutant is a potential public health threat as recent studies have revealed that the exposure to O_3_ is associated with the risk of asthma, reduced lung function, and respiratory diseases (27–29).

According to the GBD study 2010, ambient O_3_ pollution has caused ~1,440 thousand and 1,016 thousand global DALYs in male and female populations, respectively (9), whereas it caused ~400 thousand and 250 thousand DALYs in male and female populations, respectively, in China (10). The GBD study 2015 showed that COPD mortality related to global ambient O_3_ pollution has increased from 207 thousand cases in 2005 to 254 thousand cases in 2015, with a total of 22.7% increase over the past 10 years (11). Currently, the GBD studies do not include CO, NO_2_, and SO_2_ as the exposure to ambient air pollution, which might be due to the lack of suitable mathematical models and exposure data in quantifying the associations between these pollutants and disease outcome.

### Meteorological variables

Most of the ambient air pollutions are contributed by the local emission sources such as vehicle emissions and open combustion. However, other than emission factors, dispersion factors which mainly include meteorological variables such as wind speed and humidity could also significantly affect the spreading of air pollutants in ambient air (30). Adverse meteorological conditions could exacerbate the effect of regional pollution through both spatial and temporal variations, which may also increase the exposure period of harmful pollutants and contribute to an increased disease burden due to air pollution.

#### Temperature

In general, air pollutants are negatively associated with temperature, except for O_3_ concentration level which is positively correlated to temperature (31, 32). O_3_ is a secondary pollutant generated by a series of photochemical reactions between precursors such as NO_2_ and VOCs in the atmosphere. Its concentration level is predominantly affected by both precursor sources and meteorological variables such as sunlight intensity, temperature, and humidity. High ambient temperatures are often linked with noon period in a day and summer season in a year, whereas low ambient temperatures are linked with midnight period and winter season. Previous studies have shown that both high temperatures during the daytime, especially during the period of 14:00–17:00, and during the warm season, particularly from May to October, could lead to the peak O_3_ concentration level, which most likely exacerbates the ozone pollution (33).

Researchers have recently gained interests in assessing the influence of temperature on disease burden. The evidence has shown that both low and high temperatures could lead to decrements in pulmonary function, particularly in peak expiratory flow (PEF) test among COPD patients (34). Another study conducted in Tianjin pointed out a U-shaped association between temperature and mortality, which suggested that both extreme low and high temperatures increase the risk of mortality (35). Furthermore, temperature and humidity are often considered as the confounding factors in the stage of model development, which need to be carefully adjusted in the previous studies (36, 37). The prediction accuracy of the model can be significantly improved by considering the influence of meteorological variables such as temperature and relative humidity (38, 39). Furthermore, a meta-analysis reported that the respiratory mortalities increased by 0.6 and 2.1%, respectively, for each 10 μg/m^3^ increase in PM_10_ when PM_10_ was modified by low and high temperatures (40). Similarly, a study using the data of eight Chinese cities showed that 1.79% increase in respiratory mortality was observed for a 10 μg/m^3^ increase in PM_10_ in the period of high temperature (41).

The risk factors of both high and low non-optimal temperatures were first added into the GBD study 2019, contributing to 54 new pairs of risk-outcome associations. According to the GBD study 2019, there were ~1,010 thousand and 946 thousand mortalities in males and females, respectively, which were caused by the non-optimal temperatures in ambient air (12). Furthermore, the low non-optimal temperature became one of the leading risks in the older people aged above 75 years in 2019 (12). Previous studies also reported the association between extreme ambient temperatures and mortality burdens, including cardiovascular and respiratory diseases (42–44). Regions with higher PM_2.5_ concentration are more susceptible to high non-optimal temperature (45). As the exposures to ambient air pollution and non-optimal temperatures introduce a higher risk to disease burden, especially cardiovascular and respiratory diseases, it is necessary to investigate how ambient temperature modifies the effect of air pollution on the disease burden.

#### Relative humidity

Relative humidity is related to the level of moisture in the atmosphere, which is closely associated with other meteorological variables such as wind speed and precipitation. Relative humidity is also affected by geographical factor, as most of the high relative humidity levels are often observed at coastal regions. Different relative humidity levels could predominantly affect the transport, dispersion, and deposition of air pollutants, especially for particulate matter in ambient air, which eventually influence the exposure to air pollutants in a population.

Recent studies also incorporated relative humidity factor in the air pollution modeling. However, the association between air pollutants and relative humidity were not consistent, as previous studies argued that pollutant concentration levels were negatively correlated to relative humidity, whereas other studies implied that they were in positive correlation to each other. These studies showed a negative correlation between air pollutants and relative humidity (46, 47). This was justified as the high relative humidity levels were linked to the greater cloud abundance and atmospheric instability, which slowed down the photochemical processes and increase O_3_ depletion (46). In addition, previous studies conducted in Chinese cities concluded that air pollutant was positively associated with relative humidity (48, 49). Previous study identified relative humidity as the key factor in the PM_2.5_ pollution and suggested that high humidity was likely favorable for causing air pollution with high PM_2.5_ pollutant level during winters in Beijing (50). They were mainly caused by the accumulation of water-soluble/secondary components rather than primary species. Furthermore, it was suggested that high PM_2.5_ pollutant levels during summer were often correlated to high relative humidity levels (50).

The influences of air pollutants such as PM_10_, NO_2_, and SO_2_ on the emergency COPD admissions were observed to be stronger during the days with low humidity, which implied that dry air may exacerbate the symptoms in a COPD patient (51). Their study also provided the evidence in which the influences of NO_2_, O_3_, and SO_2_ tend to be greater on the cool and dry days, which further highlighted that the effect of meteorological variables such as relative humidity could modify the impact of air pollutants on emergency COPD admissions (51). A study conducted in Yangtze River Delta reported that the LC mortality increased by 6.3, 4.1, and 4.6% for each 10 μg/m^3^ increase in PM_2.5_, PM_10_, and NO_2_ on a weekly basis, respectively, in the period of relative humidity less than 73.7% (32). Previous study has reported that greater effect of air pollution on allergic rhinitis, which is one of the allergic respiratory diseases, were observed at low temperature and high relative humidity (52). Furthermore, decrease in lung function, as measured in forced vital capacity (FVC) and forced expiratory volume in one second (FEV_1_), was associated with increases in temperature and relative humidity (53). Limited evidence was given to prove the direct effect of ambient humidity on disease outcomes, instead this might motivate researchers to investigate further on the effect of relative humidity on the relationship between air pollution and disease burden.

### Disease burdens attributable to air pollution and meteorological factors

The most common disease burdens associated with air pollution are cardiovascular and respiratory diseases. According to the latest GBD study in 2019, COPD and LC were the leading causes of global mortality with respect to non-communicable diseases (NCD), followed by ischemic heart disease and stroke, making them as the major health challenges in the recent years. Both short-term (acute) and long-term (chronic) effect of ambient air pollution on disease burdens have been reported using different epidemiological study designs, including time series, cross-sectional, case-control, cohort study, and case study (54, 55).

#### Chronic obstructive pulmonary disease

Chronic obstructive pulmonary disease (COPD) is a common chronic respiratory disease characterized by chronic airway inflammation and persistent airflow limitation that is irreversible (56). COPD is not limited to a disease, but also a group of respiratory diseases that includes chronic bronchitis, emphysema, bronchiolitis, and small airways disease (56). However, other specific causes of airflow obstructions, for example, cystic fibrosis, bronchiectasis, and bronchiolitis obliterans, are not considered in patients with COPD (56). COPD can be diagnosed by identifying the ratio of postbronchodilator forced expiratory volume in the first second (FEV_1_) to forced vital capacity (FVC) that is <70% (56).

The major risk factor for COPD is associated with tobacco smoke, however, the increased risk of COPD is also related to the environmental factors, for example air pollution, temperature, and relative humidity (34, 57). Although the direct relationship between air pollution exposure and COPD is less understood, the pathogenesis of COPD is thought to be associated with oxidative stress and airway inflammation caused by the toxic pollutants (56, 58). Air pollutant, accompanied by other chemical substances, could potentially induce the production of reactive oxygen species, worsening the immune system of COPD patient in response to the airway injury caused by oxidative stress. Furthermore, the airway inflammation caused by pollutants could significantly impact on the pulmonary function in COPD patients (59). Previous study suggested that long-term exposure to PM_2.5_ could lead to adverse health effects such as decreased lung function, emphysematous lesions, and pulmonary inflammation (60). The other possible mechanisms caused by PM_2.5_ exposure, including increase of pro-inflammatory cytokines and chemokines, systemic inflammation, and increased risk of bacterial infections, were discussed in the previous paper (61).

The associations between COPD and ambient air pollutants such as PM and O_3_ were introduced in the GBD study 2010 (9), which also reported that both PM and O_3_ increase the risk of COPD mortality. According to the GBD study 2017, there were ~227.8 thousand and 178.2 thousand COPD mortalities due to the effect of ambient PM pollution and ambient O_3_ pollution in China, respectively (62). Exposure to low O_3_ concentration potentially causes airway inflammation and bronchoconstriction (58). When the O_3_ exposure is increased, it may cause exacerbation of COPD, impaired pulmonary function, increased respiratory symptoms, and an increased risk of COPD mortality (58). Previous study reviewed the COPD attributable to air pollution and reported the significant associations between short-term exposure to all six major pollutants and the risk of COPD exacerbations (59). Among all the major pollutants, the impact of O_3_ and NO_2_ have found to be stronger on the COPD patients.

The effect of non-optimal ambient temperature could also worsen the health condition of COPD patients, which may therefore contribute to an additional risk factor of COPD mortality. Cold temperature was shown to be associated with the increased risk of COPD mortality. Facial cooling is likely to induce bronchoconstriction for COPD patients under cold temperature (63). Previously published papers have also discussed the potential pathogenic mechanism of mucous hypersecretion in the cold air-induced COPD exacerbation (64). Conversely, the mechanism by which exposure to hot temperature leads to the development or exacerbation of COPD is less understood (65). However, extreme high temperature seems to adversely impact the thermoregulation system of the body, which may therefore increase the risk of COPD mortality and morbidity. The risk factors of high and low non-optimal temperatures were first included in the GBD study 2019, which has reported that the high and low non-optimal temperatures contributed to 2.3 thousand and 175.0 thousand COPD mortalities, respectively, in China (12).

#### Lung cancer

Lung cancer (LC) is characterized by the uncontrolled growth of abnormal cells or the presence of malignant derivatives in the lungs (66). Lung carcinogenesis is a complex and multistep process, including tumor initiation and progression, which also involves DNA damage and mutations (67). When carcinogens reach to bronchial epithelial cells, it has tendency to cause mutations in proto-oncogenes and tumor-suppressor genes, thus resulting in a malignant transformation (58). Carcinogens, which are generated from processes such as industrial combustion and vehicle exhaust, are often transported along with fine PM in the environment. They are likely to be responsible for the increased risk of lung cancer in most of the urban areas with high population density (68, 69). Previous studies have shown the important connection between air pollutants and LC. Air pollution exposure has been linked to the generation of reactive oxygen species and oxidative damage to DNA that might be associated with the increased risk of lung cancer (70).

Previous study conducted a study using weight-of-evidence approach to determine the evidence of a biologically plausible mode of action for PM and LC (66). The evidence supported the association between PM and DNA damage and repair, indicating the biological plausibility of mode of action. In general, the studies conducted so far have concluded that the exposure to PM might induce direct DNA damage that plausibly results in the development of lung cancer (66). Previous study analyzed the lung cancer attributable to PM_2.5_ and environmental tobacco smoke and summarized the possible mechanisms that could explain the associations (61). Exposure to PM_2.5_ seems to induce the hypermethylation of oncogene, for example, mutation of oncogene p53 is linked to the development of the LC (71). Moreover, the tumor microenvironment due to PM_2.5_ exposure may also lead to the development of LC by inducing various pro-inflammatory cytokines in the lung (61).

Although the primary risk factor for the development of LC has been shown to be associated with tobacco smoking (68, 72, 73), only 10% of smokers develop lung cancer (67). Additionally, LC in never-smokers was one of the common causes of cancer mortality (61). An increased risk of LC has been associated with other factors, for example exposure to ambient air pollution, diet, and second-hand smoke (68, 73). The studies conducted in China have shown that the prevalence of smoking was decreased in both sexes from 60.6 to 51.6% and from 4.0 to 2.9% in both males and females, respectively, in the period of 1991–2011 (74). However, the LC mortality rate of both sexes in China was found to have increased in the period of 2006–2015 (73). This might imply that the other potential factors could possibly contribute to an increased rate of LC mortality, including the increased exposure to ambient air pollution due to rapid industrialization and urbanization over the past decade. Furthermore, another study analyzed the long-term effect ambient air pollution on lung cancer and COPD mortalities in China (75). Both long-term and short-term exposures to air pollutants, including PM_2.5_, NO_2_, and O_3_, were investigated to evaluate the significant effect of air pollution on LC in the open literature. Most of these findings have clearly demonstrated that an exposure to higher level of air pollution, especially fine particulate matter, was associated with an increased risk of LC mortality.

## Relative risk model in health risk assessment

In a health risk assessment, a relative risk (RR) model provides the risk estimate that determines the risk of an adverse health impact in a population exposed to higher level of ambient air pollution as compared to that exposed to lower pollution level. The RR model could only describe certain types of adverse health outcome such as COPD and LC mortalities attributable to the effect of air pollution that happened in a defined population. The RR value of air pollution-related burden of disease was often employed to estimate the attributable mortality, and the health cost in the previous studies. These existing RR models were often recommended to estimate the RR value of air pollution exposure in the global burden assessment, as summarized in Table 1. The current study focuses on the RR models of mortality attributable to long-term ambient PM_2.5_ exposure in which its effects can often last for a longer time such as weeks, months, or years.

**Table 1:** Existing RR models used for assessing burden of disease.

**Exposure-response metric**	**RR function and its parameters used**
Long-term PM_2.5_-Cardiopulmonary mortality and lung cancer (76)	$R={\left[\left(x+1\right)/\left(x_{o}+1\right)\right]}^{\beta }\;$ *x* = pollutant concentration *x*_*o*_ = target or threshold concentration of pollutant β = coefficient
Long-term PM_2.5_-Mortality of stroke, IHD, COPD, and LC and incidence of ALRI (77)	*R* = 1 , *x* < *x*_0_ $R=1+\alpha \left(1-\exp\left[-{\gamma \left(x-x_{0}\right)}^{\delta }\right]\right)\;,\;x\geq x_{0}$ *x* = pollutant concentration *x*_0_ = counterfactual concentration of pollutant α, γ, δ = coefficient
Long-term PM_2.5_-Mortality of stroke, IHD, COPD, and LC (78)	$R=1+\alpha \times {\left(x-x_{o}\right)}^{\beta }\;$ *x* = pollutant concentration *x*_*o*_ = target or threshold concentration of pollutant α, β = coefficient
Long-term PM_2.5_-Mortality of NCD (including stroke, IHD, COPD, and LC) and LRI (79)	$R\;=\exp\left\{\frac{\theta \;log\left(\frac{z}{\alpha }+1\right)}{1+\exp\left\{\frac{-\left(z-\mu \right)}{v}\right\}}\right\}\;$ *z* = max(0, *x*−*x*_0_) *x* = pollutant concentration *x*_0_ = counterfactual concentration of pollutant θ, α, μ, *v* = coefficient

The exposure-response relationships between long-term exposure to ambient PM_2.5_ and cardiopulmonary and lung cancer mortality can be estimated using the log-linear (LL) scaling as indicated by Equation 1. The log-linear model was proposed in estimating the RR to obtain a more realistic relationship for the air pollution exposure as its slope flattens at higher pollutant concentration. The parameter *x*_*o*_, expressed as the threshold concentration of PM_2.5_, is assumed to be its background concentration which is ~5 μg/m^3^ and β is a coefficient which is specific with respect to disease outcome. There are few assumptions that need to be considered when employing these models, for example, the shape of the concentration-response function, the assumed threshold concentration, and the exposure data used in the original estimates (76).


(1) $$\begin{array}{l} R_{LL}={\left[\left(x+1\right)/\left(x_{o}+1\right)\right]}^{\beta } \end{array}$$

### Integrated exposure-response model

The use of the integrated exposure-response (IER) model for estimating RR of PM_2.5_ pollutant have been proposed for global burden of diseases, including stroke, IHD, COPD, LC in adults, and acute lower respiratory infection (ALRI) in children <5 years of age (77). The risk estimate was extended to higher concentration levels which enabled the global RR estimation over the almost entire exposure range. The IER model has been proved to be able to describe the association between RR and long-term exposure to PM_2.5_ from the four different sources: ambient air pollution, active smoking, second-hand tobacco smoke, and household air pollution (77). The IER model can be expressed by Equation 2.


(2) $$\begin{array}{c} R_{IER}=1\;,\;x<x_{0}\\ R_{IER}=1+\alpha \left(1-\exp\left[-{\gamma \left(x-x_{0}\right)}^{\delta }\right]\right)\;,\;x\geq x_{0} \end{array}$$

where *x* is the exposure to ambient PM_2.5_. α, γ and δ are the parameters that are estimated based on the data collected. As one of the important parameters of RR model, the counterfactual concentration of PM_2.5_ (*x*_0_) was often ranging from a lower limit of 5.8 μg/m^3^ to an upper limit of 8.8 μg/m^3^ in most of the studies. It is assumed that there is no health implication and *R*_*IER*_ = 1 when it is below the counterfactual concentration. *R*_*IER*_ is assumed to be 1 + α when pollutant level is very high. Moreover, *R*_*IER*_ is assumed to be 1 + αγ when concentration equals to *x*_0_ + 1.

There were five major assumptions made when developing the IER model in their study (77). Firstly, exposure to PM_2.5_ was correlated with increased mortality from stroke, IHD, COPD, and LC and with increased incidence of ALRI. Secondly, at any constant exposure level, all types of combustion sources were assumed to contribute equivalent harmful impacts which are expressed as RR. The toxicity was only dependent on the exposure but not on PM_2.5_ composition. Thirdly, the exposure-response curve between RR and PM_2.5_ was not constrained to a linear function. Fourthly, the study did not consider the temporal pattern of exposure when developing the RR of mortality from chronic disease. Finally, RR was developed for each type of exposure under the condition that there was no association between different types of exposure.

The authors have demonstrated the IER model by comparing its predictive performance with seven other models including the logarithm model and power model (77). The performance of these models was assessed by analyses and comparisons using Akaike and Bayesian information criteria (AIC and BIC), in which a model with lower values of AIC and BIC provided a better fit than other. Generally, the results have indicated that the proposed IER model can match well the RR for all the PM_2.5_ sources and causes of mortality, however, it does underestimate the RRs for the association between COPD and household air pollution. In fact, the power model can fit better for the COPD mortality with the lowest AIC and BIC values obtained. The authors also compared the RR estimates obtained from the IER model with that obtained based on the Chinese cohort for the three health outcomes (mortalities of IHD, stroke and LC). The result, which was done by a similar calculation, has clearly indicated that the IER model can provide reasonable estimations of RR values in the regions with higher pollutant level such as China (77). Nevertheless, the comparison analysis of RR estimates using the IER model for COPD mortality estimation in the more polluted regions was not presented and the conclusion was unclear.

Previous studies that applied the IER model in the analysis of ambient air pollution-related disease burden only focused on four specific diseases, which are IHD, stroke, COPD, and LC, because the development of the IER model relies to a great extent on the available RR information of these diseases. The IER model was increasingly used to estimate the attributable mortality using the result of calculated RR. Previous study has evaluated the global PM_2.5_ concentration-mortality relationships by using the IER model outputs (80). By applying the estimated relative risk (*R*), the premature mortality (*M*) for a specific disease outcome in a population is measured using Equation 3, with *P* and *I* indicating population and regional average annual disease mortality rate (also known as baseline mortality rate), respectively (80).


(3) $$\begin{array}{l} M=P\times I\times \left[1-\frac{1}{R}\right] \end{array}$$

Since the IER model was constructed based on mostly the available RR information of the regions with lower pollution level such as North America and Europe, the performance of IER model remains uncertain in the regions with higher pollution level such as China. Previous study conducted a comparison analysis between the IER model and a new concentration-response model called shape constrained health impact function (SCHIF), which was developed by a cohort study in China (81). The result indicated that IER model was likely to underestimate the health impact at higher level of PM_2.5_ level, especially for stroke and COPD (81). This might also imply that the IER model is less suitable for the estimation of PM_2.5_-related disease burden in the more polluted regions such as China.

The curve of IER model with respect to different health outcome was not a linear function due to the non-linear property of the model. The shape of IER model depends on the values of the unknown parameters, which may eventually result in sublinear, near-linear, supra-linear, and sigmodal curves for the association between air pollutant and mortality (77). The non-linear property of the IER model plays an important role in estimating the health risk caused by air pollution exposure as the inappropriate assumption of a linear concentration-response function up to highest observed concentration of exposure level would generate an inappropriate estimation (76). As the IER model might perform a less accurate estimation of RR in the more polluted regions such as China, especially for COPD, it is suggested to apply the long-term cohort studies for obtaining a more accurate shape of exposure-response curve in the more polluted regions (80).

Despite the researchers having attempted to include the studies of high PM_2.5_ from other exposures such as tobacco smoking in the IER model, the lack of cohort studies with high ambient PM_2.5_ has made the IER model unfavorable in estimating a more accurate value of RR in the more polluted regions. Therefore, the shape of current IER model remains unclear in the high ambient PM_2.5_ level, especially for COPD in which the IER model did not present the COPD result in the comparative analysis with China cohort studies. In order to produce a reasonable estimation of RR in China, it is necessary to construct a new RR model which includes more cohort studies with higher ambient PM_2.5_ level.

### Non-linear power law model

The non-linear power law (NLP) model, as shown in Equation 4, was established to estimate the RR for four cause-specific mortalities attributable to long-term ambient PM_2.5_ exposure such as IHD, stroke, COPD and LC (78). The two estimated constants, α and β are expected to be unique for different types of premature death. In their study, the parameter *x*_*o*_ was taken as 5.8 μg/m^3^ as it was the lower limit of counterfactual concentration previously used in many studies. There were three main assumptions made when generating the result in their study. Firstly, RR did not change with PM_2.5_ composition. Secondly, there was no overlap of the premature mortality attributable to PM_2.5_ exposure when calculating the total amount of annual premature mortality due to COPD, IHD, stroke, and LC. Thirdly, the effect of co-morbidity was negligible on the disease-specific risk functions. Their study also compared the estimated annual premature mortality from ambient PM_2.5_ exposure in India by employing the NLP model and the IER model. However, the performances of both NLP and IER models were not clearly compared and justified in the study (78), which may require further investigations in this research area. In other words, the study is still inconclusive when selecting the best exposure-response model to estimate both the RR and its attributable mortality.


(4) $$\begin{array}{l} R_{NLP}=1+\alpha \times {\left(x-x_{o}\;\right)}^{\beta } \end{array}$$

Another study studied the PM_2.5_-related burden of diseases and its economic impact in 338 Chinese cities (4). The premature mortality attributable to PM_2.5_ exposure was estimated and compared using the IER model, the NLP model, and the LL model in their study. Their study used the PM_2.5_ threshold concentration of 5.9 μg/m^3^ for the RR estimates. The total premature mortality attributable to PM_2.5_ in China estimated using the LL model was found to be the highest (1.258 million), followed by those using the IER model (0.964 million) and the NLP model (0.770 million). The total mortality attributable to PM_2.5_ exposure estimated by the NLP model is lowest as compared to the other two RR models, which is 25.3% lower than IER estimate. However, the NLP model is likely to have a greater estimated value than the IER model for both COPD and LC. The distribution of cause-specific mortality with respect to total estimated mortality in the NLP model is not consistent with the IER and LL models. High uncertainty for premature mortality estimated by applying the NLP model was noticed in their study. Furthermore, the application of using different relative risk models was found to yield the uncertainty in mortality estimation attributable to PM_2.5_ exposure. It should be mentioned that these authors did not reach the conclusion in their study what the most suitable type of RR models for China should be (4), as it required more robust health data and cohort study for the analysis of RR models. Nevertheless, by using the result of mean estimate values, the NLP result was observed within most of the uncertainty range of the IER result, but not within that of the LL result which tends to be overestimated especially when PM_2.5_ exposure is at higher level. The current literature regarding the application of the NLP model in the air pollution-related health burden analysis is very limited and requires further investigation.

### Global exposure mortality model

The global exposure mortality model (GEMM) was constructed as an updated version of the log-linear (LL) model that also captures the non-linear property and relaxes some of the assumptions made when developing the IER model. Previous study has studied the hazard ratio predictions and compared it with the GBD study 2015 by using the hazard ratio model and method of statistical inference (79). More importantly, the authors addressed some of the limitations of previous IER model, as well as the improvement work that was done in their study. Firstly, there was a lack of direct evidence supporting the performance of the IER model at higher PM_2.5_ exposure level. Most of the cohort studies that studied ambient PM_2.5_ pollution in the previous study (77) were limited to regions with low exposure level, which was <35 μg/m^3^ (2). Secondly, the IER model applied the PM_2.5_ information that were based on the exposure levels on non-ambient PM_2.5_ sources such as second-hand smoking, household air pollution, and active smoking. The assumption in which toxicity of different sources of PM_2.5_ is only function of mass concentration could contribute to an instable prediction of RR value. Thirdly, the application of the IER model requires the value of counterfactual concentration of PM_2.5_, which was defined as a uniform distribution bounded by the minimum (2.4 μg/m^3^) and fifth (5.9 μg/m^3^) percentiles of exposure levels based on the cohort studies of relatively low concentration (2, 79). However, this counterfactual distribution may not accurately describe the curve of the IER model at low concentrations with the lack of direct evidence.

To enhance the prediction of disease burden attributable to long-term exposure to ambient PM_2.5_, the GEMM has been constructed based on the estimates from 15 within-cohort studies and 26 additional cohort studies that the association of ambient PM_2.5_ and mortality was both analyzed and studied. The 15 within-cohort studies, including the cohort studies in China, were proven to be able to improve the generalizability of the relative risk model. It was also found that the assumption which was based on the linear association between PM_2.5_ exposure and mortality may be relaxed for the analyses using the 15 within cohorts. However, the study on the 26 additional cohorts has assumed a linear relationship between PM_2.5_ exposure and the logarithm of the baseline hazard ratio due to limited access to the subject level information (79). It should be pointed out, moreover, that the within-cohort studies only focused on non-accidental mortality, which was mostly caused by non-communicable diseases (NCD) and lower respiratory infections (LRI). Both non-accidental mortality (GEMM NCD + LRI) and sum of five cause-specific mortalities (GEMM 5-COD) were used as the outcomes to quantify the mortality burden attributable to ambient PM_2.5_ in 2015 and compared with the estimation with the original IER model (79). A simplified version of the GEMM can be also found in their paper, as shown by Equation 5. Additionally, the values of unknown parameters such as θ, α, μ, and *v* have been suggested in their paper.


(5) $$\begin{array}{lll} R\; & = & \exp\left\{\frac{\theta \;log\left(\frac{z}{\alpha }+1\right)}{1+\exp\left\{\frac{-\left(z-\mu \right)}{v}\right\}}\right\}\\ z & = & \max\left(0,x-x_{0}\right) \end{array}$$

As the GEMM was recently developed, there is a growing number of studies that have attempted to apply it for the estimation of ambient air pollution-related disease burden. Previous study has compared the performance of the GEMM with the IER model and the LL model using the PM_2.5_ concentration in China, and estimated the attributable mortality (82). It can be clearly seen that the estimated mortality of GEMM NCD + LRI is notably higher than other models, which may imply that the GEMM is able to estimate the attributable mortality of the diseases other than these five major diseases. The authors demonstrated that the use of the IER model and the GEMM produces the results of estimated 0.96 million and 1.60 million of ambient PM_2.5_-attributable mortality in China in 2015, respectively (83). By using the GEMM, it was found that there is an average 63% higher PM_2.5_-attributable mortality in China as compared to the IER model. Another similar study (84), which has conducted the estimation of mortality attributable to ambient PM_2.5_ in urban China under the lockdown scenario due to the pandemic 2020, has reported that a higher mortality can be obtained based on the estimation of the GEMM as compared to the IER model. In general, a higher estimated PM_2.5_-related mortality is observed in the GEMM. This may contribute to the other NCD in which the IER model did not capture. Another possible explanation is the underestimation of PM_2.5_-related mortality in the previous health risk assessment using the IER model, especially in the more polluted region such as China.

However, another study commented that the highest observed PM_2.5_ exposure among the cohort studies in GEMM was only 84 μg/m^3^ and therefore the IER model would be more suitable for the estimation of PM_2.5_-attributable mortality in China as compared to the GEMM (85). Based on their study, the maximum exposure limits of the 41 cohort studies considered in the development of GEMM were ranging from 8 to 33 μg/m^3^, except 41.9 μg/m^3^ in Asia, 49.7 μg/m^3^ in US, and 83.7 μg/m^3^ in Asia. Despite the GEMM employing all cohort studies that have provided the RR information attributable to the ambient PM_2.5_, the insufficient information of high ambient PM_2.5_ level would also make the GEMM unfavorable in estimating the RR in the regions with higher PM_2.5_ level such as China. Furthermore, out of 41 cohort studies, there was only one Chinese cohort study (86) that had included the analysis of ambient air pollution in China mainland, which might also limit the generalizability of the GEMM in the estimation of health impact attributable to ambient PM_2.5_ in China. Therefore, it would be difficult to conclude which the best RR model is for estimating ambient PM_2.5_-attributable mortality in China based on the previous studies. It is therefore necessary to conduct more advanced research to further improve the performance of the RR model, especially in the regions with higher ambient air pollution level.

## Limitations of the existing relative risk models

Most of the recent relative risk models have been related to long-term exposure to ambient PM_2.5_. However, there are many limitations involved in the existing relative risk (RR) models when assessing the burden of disease attributable to long-term ambient PM_2.5_ exposure. For example, the RR estimation using the IER model is only applicable to PM_2.5_ exposure, which is considered as the major pollutant in the assessment of health burden. However, from the previous studies, exposure to other pollutants such as CO, NO_2_, and O_3_ can also be linked to adverse health outcomes (17, 19, 87). Other major pollutants and meteorological factors such as temperature and relative humidity should be considered during the development of air pollution-related RR model. Both high and low non-optimal temperatures were previously shown to be associated with the disease burdens such as COPD (65), and their associations were also reported in the GBD study 2019 (12, 45). Furthermore, the associations between the meteorological factors such as temperature and air pollution are not uncommon and reported in the previous studies (31, 88). Therefore, it is recommended to consider the effect of ambient temperature in the development of RR model in the future studies.

So far, most of the RR used in the health risk estimation are based on epidemiological studies available for a specific health outcome. However, due to the lack of direct epidemiological evidence, especially in the more polluted regions, it is important to conduct the studies in other part of the world. Furthermore, the derivation of the IER model was based on mostly the estimates of active smoking and second-hand tobacco smoke from the North American and European cohort studies (77). This may produce potential uncertainty error for the studies related to ambient air pollution-related health burden, as well as the studies in other countries of the world. A recent cohort study conducted in China has clearly indicated that the calculated risk estimate is consistently higher than the result estimated using the IER model (86), which has suggested that the IER model may underestimate the RR values in the more polluted regions in China (89).

Both IER model and GEMM were developed based on mostly the evidence of the studies in the regions with lower PM_2.5_ exposure such as North America and Europe. Although the recently proposed GEMM has considered and included a cohort study conducted in China mainland (86), which the only study out of 41 cohort studies that measured the outdoor air pollution-related health burden in the regions with low pollutant level has been made, it is still insufficient to provide the information of exposure-response curve at the high PM_2.5_ level. The performance of the existing RR models in the estimation of mortality attributable to ambient air pollution in China are compared and summarized in Table 2. In order to provide more reasonable RR estimation in China and other more polluted regions in the world such as India and Pakistan, it is necessary to develop a more advanced exposure-response model that addresses the limitations of the existing RR models. The improvement on the RR model would be beneficial to the future studies if employing the concentration-response model derived based on Chinese cohort studies (90–92).

**Table 2:** Performance of the existing RR models in the estimation of air pollution-related mortality in China.

**Performance**	**IER**	**NLP**	**GEMM**
PM_2.5_ source	Ambient air pollution, active smoking, second-hand tobacco smoke, and household air pollution	Ambient air pollution	Ambient air pollution
Disease burden	Mortality of COPD, IHD, stroke, and LC; Incidence of ALRI	Mortality of COPD, IHD, stroke, and LC	Mortality due to NCD + LRI and 5-COD (including COPD, IHD, stroke, LC, and LRI)
Inclusion of Chinese cohort study	No	No	Yes
Inclusion of meteorological factors	No	No	No

## Concluding remarks and recapitulation

The effective estimation of ambient air pollution-related mortality is increasingly becoming important and crucial from perspective of improving our human life quality in the world, as more countries are currently experiencing the industrialization and urbanization that contribute to a more severe global air pollution. In China, higher pollutant levels are often observed in the more urbanized and industrialized regions such as Yangtze River Delta. The relative risk (RR) models have been employed in the ambient air pollution-related health risk assessment to accurately estimate the attributable mortality in the world, which leads to the importance of constantly developing a more advanced RR model in quantifying the relationship between air pollution exposure and disease burden. Previous RR models in the ambient air pollution-related health risk assessment such as the integrated exposure-response (IER) model, the non-linear power law (NLP) model, and the global exposure mortality model (GEMM) were critically reviewed to provide a comprehensive understanding on the current status of mathematical modeling in the ambient air pollution-related health risk assessment. Based on the previous studies employing the IER model and the GEMM, the most common counterfactual level of PM_2.5_ exposure was found to be ranging from 2.4 to 8.8 μg/m^3^. Nevertheless, further work should be carried out to support the evidence as to whether it is appropriate to employ the current counterfactual level in the RR models, especially in the more polluted regions such as China.

Furthermore, the limitations of previous RR models were studied and summarized as follows: (1) Ambient PM_2.5_ is the only ambient air pollutant of interest in the most of the previous RR models. The previous RR models also did not include the effect of meteorological factors such as temperature, relative humidity, and wind speed, which were shown to have significant impact to the transport and dispersion of air pollution in ambient air. Both high and low non-optimal temperatures were proven to be associated with a higher risk of mortality such as COPD in the GBD study 2019. There is a need to update the existing RR model by incorporating the effect of meteorological factor such as temperature. (2) The existing RR models, including the IER model and the GEMM, were developed based on most of the studies that were conducted in the regions with low ambient air pollution level such as North American and European countries. Although the GEMM has included a recent Chinese cohort study (86), there are significant uncertainties attached to the shape of RR model at higher ambient PM_2.5_ level and particularly to the mortality estimation in the health risk assessment in China. It is suggested to employ evidence from the Chinese cohort studies when developing a more promising RR model for the estimation of disease burden attributable to ambient air pollution in China. (3) Other than ambient air pollution, the development of the IER model also relies on the information from other exposures such as active smoking, second-hand tobacco smoke, and household air pollution. In general, the exposure to active smoking is exceedingly higher than the exposure to ambient air pollution, which therefore considerably controls the shape of the exposure-response curve at higher PM_2.5_ exposure. These might lead to significant uncertainties related to the estimation of mortality attributable to ambient air pollution and the shape of the IER model at higher ambient PM_2.5_ level. It is necessary to apply the evidence based on solely the studies of ambient air pollution when developing a more suitable RR model. In the future research of this aspect, one of the focuses is the development of an advanced RR model, which incorporates the effects of ambient PM_2.5_ and the meteorological factor such as temperature, for the estimation of disease burden such as COPD and LC mortalities in the more polluted regions such as China.

The modeling of PM_2.5_-related mortality remains a substantial challenge owning to the uncertainties and limitations of the previous RR models. As shown in Equation 3, other than a suitable RR model, the mortality estimation also involves other important factors such as PM_2.5_ exposure, population distribution, and baseline mortality. The difference in RR models could contribute to a major part of uncertainties in the estimation of mortality attributable to ambient air pollution, which may potentially affect the policy making decision of the health authorities. Furthermore, the estimation of health cost related to ambient air pollution-related mortality, including the economic loss due to disease burden, also significantly depends on the RR model. Being one of the most important steps in the health impact assessment, the RR model should be aimed to provide a more realistic estimation of health burden, which is highly relevant to air pollution-related policy measures, especially when planning the most suitable control strategies to achieve air pollution reduction. As China is the largest country by population and one of the most industrialized countries in the world, it is essential to develop a more suitable and reliable RR model that can provide a more accurate estimation of ambient air pollution-related disease burden in the country.

## Author contributions

CC: conceptualization and writing—original manuscript preparation. JY: supervision and writing—language and proofreading. XY: supervision, conceptualization, and writing—reviewing and editing. JH: supervision and data curation. All authors contributed to the article and approved the submitted version.

## Funding

This study was financially supported by the National Natural Science Foundation of China (Grant No. 21750110446).

## Conflict of interest

The authors declare that the research was conducted in the absence of any commercial or financial relationships that could be construed as a potential conflict of interest.

## Publisher's note

All claims expressed in this article are solely those of the authors and do not necessarily represent those of their affiliated organizations, or those of the publisher, the editors and the reviewers. Any product that may be evaluated in this article, or claim that may be made by its manufacturer, is not guaranteed or endorsed by the publisher.

## Abbreviations

AIC: Akaike information criteria
ALRI: acute lower respiratory infection
BIC: Bayesian information criteria
COPD: chronic obstructive pulmonary disease
DALY: disability-adjusted life year
GBD: global burden of disease
GEMM: global exposure mortality model
IER: Integrated exposure-response
IHD: ischemic heart disease
LC: lung cancer
LL: log-linear
LRI: lower respiratory infections
NCD: non-communicable diseases
NLP: non-linear power law
PM: particulate matter
TBL, tracheal: bronchus and lung
VOC: volatile organic compound.
